# Soil depth exerts greater effect on bacterial community than spatial structure in Longmenshan fault zone

**DOI:** 10.1128/aem.01161-24

**Published:** 2025-03-12

**Authors:** Peng Jiang, Xin Wan, Mingxuan Che, Lihuan Li, Mingxue Liu

**Affiliations:** 1 School of Life Science and Engineering, Southwest University of Science and Technology 644874 , Mianyang, Sichuan, China; 2 College of Wuliangye Technology and Food Engineering, Yibin Vocational and Technical College 562725 , Yibin, Sichuan, China; Colorado School of Mines, Golden, Colorado, USA

**Keywords:** bacterial, soil depths, spatial structure, keystone species, co-occurrence network, mountainous

## Abstract

The diversity patterns and drivers of soil microbial communities across spatial distances have been extensively investigated over the recent years. However, whether microbial communities in surface and subsurface soils showed an identical spatial distribution pattern at a small regional scale has not been fully confirmed. For this, we investigated the linkage between soil water content (SWC), pH as well as nutrient contents and soil bacterial diversity and communities in different soil layers in the Longmenshan fault zone in Sichuan Province, China. Our investigation indicated that surface soil bacterial communities were positively correlated with SWC and pH (*P* < 0.05), whereas those in the subsurface correlated with soil organic carbon and its fractions (*P* < 0.05). Bacterial community co-occurrence network structures differed significantly across soil layers. Compared to subsurface soils, surface soils had more nodes, larger network diameters, and longer average paths. The keystone species belonged to Rokubacteriales in the surface, and in the subsurface soil, they belonged to Chryseobacterium, while no keystone species were found in the subsoil. Spatial distance showed a smaller effect (4%–7%) on community structure, indicating that various soil factors represent key regulatory factors for bacterial community shifts. Collectively, soil depth showed a considerably higher effect than spatial distance on soil bacterial community composition and network properties in Longmenshan fault zone, with key species differing across soil layers. These results could provide an essential basis for further investigation of microbial functions in soil ecosystem heterogeneity and maintaining soil health.

**IMPORTANCE:**

Soil water content served as the main driver of changes in surface soil bacterial diversity and community. Spatial structure had a greater influence on surface soil bacterial communities and diversity. Soil depth had a significantly higher effect on soil bacterial community composition and network properties than site. Our results may provide insights into the impact of microbial functions on biodiversity and ecosystem service functions.

## INTRODUCTION

Mountain ecosystems represent an important component of terrestrial systems and provide numerous ecological services (1–3). Mountain ecosystems are characterized by high variability in climate, vegetation, and soil properties over short spatial distances and altitudinal gradients (4, 5). And these variations could affect soil microbial community structure and functions (6–9). Microorganisms are important components of mountain ecosystems and play a key role in biogeochemical cycling, material and energy transformation, and maintaining the stability of ecosystems (6–8, 10–12). Thus, investigating the drivers of microbial community structure in mountain ecosystems is important.

Obviously, soil microbial diversity and communities closely depend on soil properties (6, 7, 9, 10, 12, 13). Variations in soil properties may result in different ecological processes as well as variable degrees of environmental filtration, causing differences in microbial communities (6, 7, 14). The soil physicochemical properties (e.g., pH and nutrient availability) that control soil nutrient cycling have been shown to affect microbial community structure (12, 15). In Changbai Mountain surface soils, pH was the major factor influencing bacterial diversity, whereas in deep soils, carbon effectiveness was the main driver (16). Analogous studies have shown that soil organic carbon (SOC) content affects microbial diversity in surface soils, whereas microbes in deep soils are affected by the carbon component (7). Also, microbial communities may be more stable in deep soils as they are less affected by the external environment (15, 17). Thus, the effects of soil properties on microbial community probably varied considerably.

Soil microbial communities have been demonstrated to be strongly influenced by spatial structural heterogeneity at the global scale. Soil properties affect microbial communities with considerable uncertainty, which may be due to variations in soil type, vegetation, and climate at the various study sites (5, 14). At the regional scale, however, the relative contribution of spatial structure to variation in soil microbial communities remains unclear. Several studies identified soil characteristics as more influential on shifts in bacterial communities than geographic distance and climatic factors (18, 19). Also, some studies found that soil microbial community composition correlated not with spatial structure (5, 20). The studies indicated that soil properties and SOC vary considerably across soil layers; however, knowledge of whether spatial factors affect deep soil bacterial communities remains limited. Thus, investigations into the factors affecting the variability of soil microbial community structure at a small scale could be very valuable.

Our study analyzed the bacterial diversity and the relationship between community structure and environmental characteristics in 15 sample plots at five sampling sites within the northern mountainous area of the Longmenshan fault zone. We assumed that the bacterial diversity in surface soils would be significantly higher than in subsurface soils, given that the content of soil organic matter (SOM) decreases with soil depth. Based on this, combined with the results of previous studies, we hypothesized that (i) soil bacteria community diversity variation across sites at small scales is insignificant due to small climatic variations and consistent vegetation, (ii) soil bacterial community was driven variously across soil layers due to interactions of environmental and resource competition or diffusion barrier variability, and (iii) bacterial networks varied across soil layers and key species differed varied.

## MATERIALS AND METHODS

### Study sites

**Fig 1:**
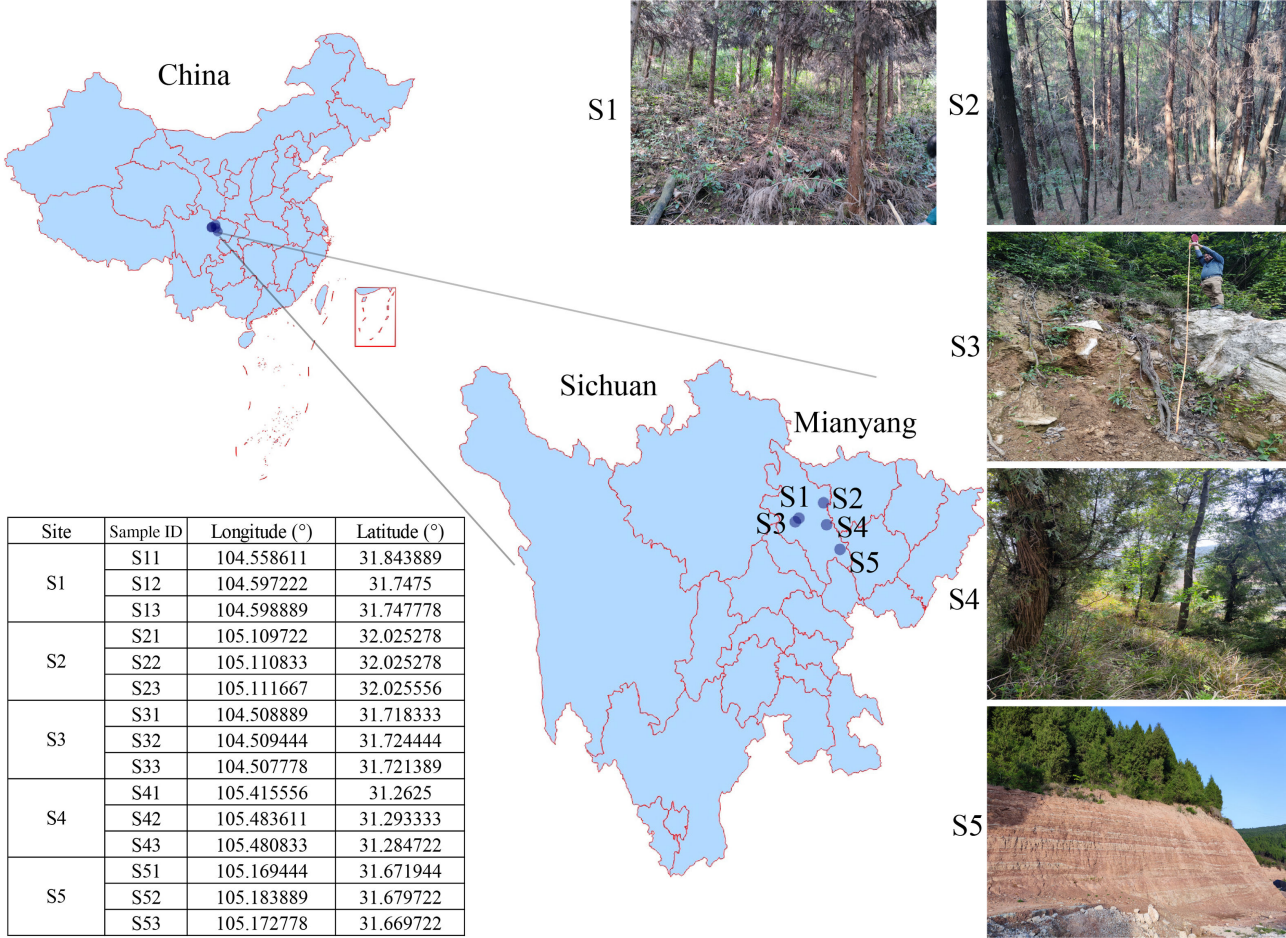
Site locations of the study area. The maps were created using the Biozeron Cloud Platform (22).

The study was carried out in Mianyang City, Sichuan Province, southwestern China (31°15′50″–32°14′16″N, 104°30′28″–105°28′51″E) (Fig. 1). The region lies in the northern part of the natural boundary between the Sichuan Basin and the western Sichuan Plateau and is also the seismic activity zone on the rim of the Qinghai-Tibetan Plateau (15, 21). The region belongs to the subtropical wet monsoon climate, the average annual temperature ranges from 14.7°C to 17.3°C, and the annual precipitation is from 825.8 to 1,417 mm. The dominant vegetation types in the area are *Cupressus funebris* Endl., *Alnus cremastogyne* Burkill, *Robinia pseudoacacia* L., *Quercus aliena* Blum, *Quercus palustris* Münchh., and *Imperata cylindrica* (L.) P. Beauv.

### Experimental design and sampling

Sampling to be conducted during the spring of 2022. Five sampling places were selected in our study area (Fig. 1). Three 10 × 10 m sampling sites were randomly selected for each sample place. Three profiles with a depth of 50 cm were set up at each sampling site, and soil samples were collected at 0–5 cm, 5–25 cm, and 25–50 cm in each profile, labeled as T, M, and S, respectively. The soil samples from the sampling sites were then mixed homogeneously, and a total of 45 mixed samples were collected. Finally, the mixed samples were divided into three parts: one was stored at −80°C for amplicon sequencing, another was placed at 4°C for microbial biomass carbon (MBC) determination, and the rest was air-dried indoors for soil physical-chemical properties.

### Determination of soil characteristics

SOC was determined by the potassium dichromate oxidation method (23). Readily oxidizable organic carbon (ROC) content was determined using the KMnO_4_ oxidation method (24). The samples were dried in an oven at 105°C for soil water content (SWC) determination, and pH was measured by pH meter (water:soil = 2.5:1). Cold water extractable organic carbon (CEOC) and hot water extractable organic carbon (HEOC) were extracted with a 1:4 mixture (soil:water ratio) (25, 26). MBC was determined by chloroform fumigation (27). Soil ammonium nitrogen (NH_4_^+^) and nitrate nitrogen (NO_3_^−^) were determined by the colorimetric method (5, 28).

### Soil DNA extraction and Illumina sequencing

DNA was extracted from soil using FastDNA Spin Kit (MP Biomedicals, Santa Ana, CA, USA). The V3-V4 hypervariable region of 16S rRNA gene was amplified using the primers 338F (5′-ACTCCTACGGGAGGCAGCAG-3′) and 806R (5′-GGACTACHVGGGTWTCTAAT-3′) (8). Amplification of the 16S rRNA gene by PCR was performed by thermal cycling PCR systems (29, 30). PCR products were further purified and quantified using Agencourt AMPure Beads (Beckman Coulter, Indianapolis, IN, USA) and PicoGreen dsDNA Assay Kit (Invitrogen, Carlsbad, CA, USA). Finally, sequencing was performed at Shenzhen Huada Gene Technology Co., China.

### Statistical analysis

All values were denoted as the mean ± standard deviation. Data were log-transformed to satisfy the assumptions of the parametric tests. Two-way nested analysis of variance (ANOVA) was utilized to analyze the effects on soil properties and bacterial communities across sites and soil layers. Non-parametric multivariate statistical tests (analysis of similarities [ANOSIM] and permutational multivariate analysis of variance [ADONIS]) and non-metric multidimensional scaling (NMDS) analyses were applied to identify the differences in bacterial community across soil sites and layers. Spearman’s rank correlation analysis and stepwise multiple regression were performed to investigate the effects of soil properties on the bacterial community. Taxonomic distances for bacterial communities were analyzed using Bray–Curtis and Euclidean distances in partial Mantle tests (5).

Non-randomized co-occurrence network methods were used to elucidate bacterial interactions in different soil layers and sites (31, 32). Correlation coefficients were limited to correlations greater than 0.7 or less than −0.7 (*P* < 0.05). Visualizations were performed with Gephi 0.9.7 using the Fruchterman Reingold layout. The “igraph” package was utilized to acquire the subnetwork topology network properties, and the network nodes were defined using the method proposed by Song et al. (4). Zi and Pi values were calculated using the “igraph” package. Two-way ANOVA was used to determine the effect of site and soil layer on the network topological properties. Spearman’s correlation coefficients were calculated to assess the relationship between network properties and environmental parameters and to assess the effect of different biogeographic parameters on co-occurring network parameters.

The relationship between soil factors and bacterial communities was investigated using the constrained redundancy analysis (RDA) (5). Variation partitioning analysis was performed to distinguish the effects of environmental factors on the bacterial community using the “Vegan” package. The environmental factors are divided into two categories: (i) soil factors, i.e., the soil properties measured in this paper and (ii) spatial structure as calculated in references 5, 33.

## RESULTS

### Variation in soil characteristics

In our study, site and soil layers had a considerable effect on soil characteristics (Table 1; also see Fig. S1 at https://doi.org/10.6084/m9.figshare.28255817.v1). Overall, the site showed a clear effect on pH, SWC, SOC, ROC, DOC, NO_3_^−^, NH_4_^+^, HEOC, CEOC, and MBC. Soil layer had a significant effect on all of these soil properties other than pH value. SWC, SOC, MBC, and NO_3_^−^ decreased with soil depth. Averages of SOC, NH_4_^+^, and MBC content in topsoil layer were 26.72 g/kg, 11.06 mg/kg, and 250.63 mg/kg more than those in the deeper soil layers, respectively. The MBC content in the deeper soil layer reduced by more than 50% from the topsoil layer. Also, these soil properties showed distinct variability patterns across sites.

**TABLE 1:** Two-way nested ANOVA testing the sites and soil layers (nested within sites) on soil characteristics

	Df	Sum Sq	Mean Sq	*F* value	Pr (>*F*)^*a*^
Site	4	0.130	0.033	5.313	<0.01**
Site (layer)	10	0.727	0.073	11.854	<0.01**

^*a*^ **, *P* < 0.01.

### Bacterial α-diversity and community composition

**Fig 2:**
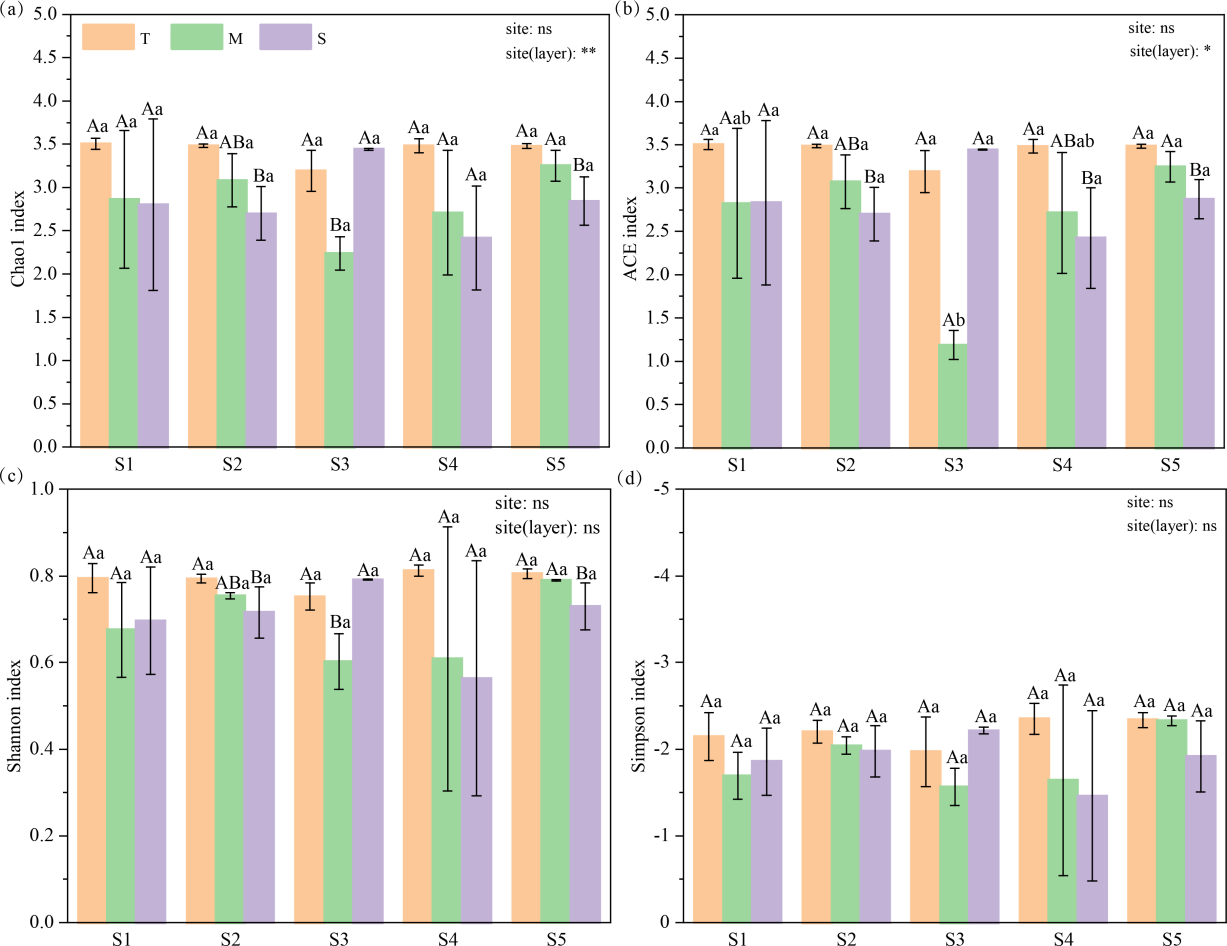
Chao1 index (**a**), ACE index (**b**), Shannon index (**c**), and Simpson index (**d**) of bacterial communities across layers. “*”, “**”, and “ns” indicate the significant levels for sites and soil layers (nested within sites) at 0.05, 0.01, and nonsignificant, respectively. Upper case letters varied to indicate significant differences (*P* < 0.05) across soil layers at the same site and lower case letters varied to indicate significant differences (*P* < 0.05) among sites in the same soil layer.

A total of 2,648,380 valid sequences were obtained from the 16S rRNA gene sequences of the soil samples (45 samples) and were clustered at a genetic distance of 0.03 into 62,613 operational taxonomic units (OTUs) at the 97% similarity level belonging to 39 phyla, 103 classes, and 927 genera of the bacteria in our study. And we applied bacterial community richness (Chao1 and ACE indices) and diversity (Shannon and Simpson indices) as indicators of bacterial α-diversity. The distribution of soil bacterial community diversity decreased with soil depth in our study (Fig. 2; also see Fig. S2 at https://doi.org/10.6084/m9.figshare.28255817.v1). Soil layer had a significant effect on the indicators of α-diversity with Chao1 and ACE index, whereas the site did not significantly affect α-diversity.

**Fig 3:**
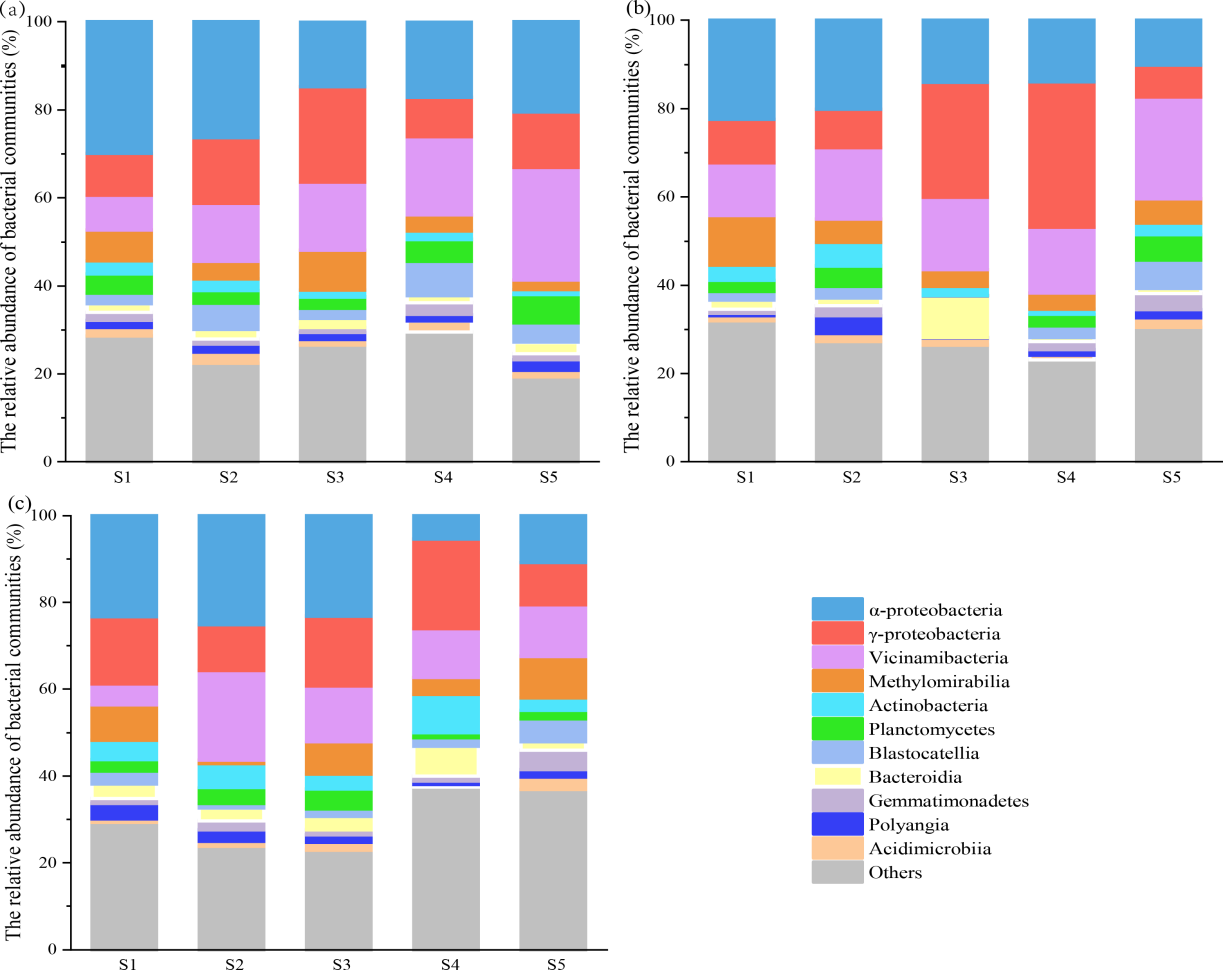
The relative abundances of dominant soil bacterial groups at the class level at T (**a**), M (**b**), and S (**c**) layers.

The dominant bacterial classes in collected soil layers (T, M, and S) were α-Proteobacteria, γ-Proteobacteria, and Vicinamibacteria (relative abundance > 5%), which together accounted for roughly 40% across sites (Fig. 3). α-Proteobacteria and γ-Proteobacteria are members of Proteobacteria, and Vicinamibacteria is one of the Acidobacteria. As such, the bacterial community consisted mainly of Proteobacteria and Acidobacteria across soil layers. NMDS based on the Bray–Curtis distance revealed obvious variations in bacterial community across soil layers (see Fig. S3 at https://doi.org/10.6084/m9.figshare.28255817.v1). The permutational multivariate analysis of variance (PerMANOVA) further verified that obvious deviations in bacterial composition were present across sites and soil layers (see Fig. S3 and Table S1 at https://doi.org/10.6084/m9.figshare.28255817.v1). Both site and soil layer had an important effect on relative abundance of Gemmatimonadetes, Blastocatellia, Planctomycetes, and α-Proteobacteria (see Fig. S4c, e, f, l at https://doi.org/10.6084/m9.figshare.28255817.v1).

### Effects of environmental variables on bacterial α-diversity and community

The Simpson and Chao1 indices in the T layer were positively and negatively related to SWC, respectively, and the Shannon index was negatively correlated with NO_3_^−^ (Table 2). There was no significant correlation between different soil factors and Chao1 index (ACE index). In the M layer, Chao1, ACE, and Shannon indices were most strongly positively correlated with DOC (Table 2). Notably, the Shannon index was positively correlated with pH. In the S layer, Chao1, ACE, and Shannon indices were most positively correlated with ROC and CEOC (Table 2). In the T layer, multivariate stepwise linear regression results showed that SWC was the main contributor to the Shannon index (34.4%) and Simpson index (33.6%) (see Table S2 at https://doi.org/10.6084/m9.figshare.28255817.v1). In the M layer, ROC was the main variable contributing to variation in bacterial Simpson (40.1%) and Chao1 (43.9%), respectively. DOC was the main contributor to the Shannon index (34.0%). In the S layer, SOC was the main contributor to the Shannon index (34.4%) and Simpson index (33.6%). ROC, DOC, and SOC were the main variables contributing to variation in bacterial richness and diversity. Thus, SWC contributed the most to variation in bacterial diversity in the T layer, while SOC and its fractions were the dominant variables contributing to bacterial richness and diversity in the M and S layers.

**TABLE 2:** Spearman’s rank correlations (*r* value) between richness and diversity of bacterial communities at class level with soil characteristics across soil layers^*a*^

Plot	Index	pH	SWC	SOC	ROC	MBC	HEOC	CEOC	DOC	NH_4_^+^	NO_3_^−^
T	Simpson	−0.482*	0.514*	0.393	0.421	0.207	0.079	−0.011	0.064	0.436	0.225
	Chao1	−0.107	−0.207	−0.021	0.041	0.161	0.171	0.321	0.264	−0.143	−0.243
	ACE	−0.111	−0.193	−0.011	0.025	0.154	0.164	0.325	0.254	−0.132	−0.221
	Shannon	0.329*	−0.532*	−0.379	−0.404	0.018	−0.050	−0.061	0.007	−0.450	−0.607*
M	Simpson	−0.590*	0.473	−0.077	−0.253	0.049	−0.549	−0.621*	−0.687**	0.190	0.214
	Chao1	0.232	−0.176	0.577*	0.703**	0.060	0.731**	0.297	0.753**	0.152	−0.126
	ACE	0.234	−0.225	0.577*	0.709**	0.082	0.714**	0.231	0.725**	0.138	−0.132
	Shannon	0.629*	−0.484	0.242	0.352	0.016	0.610*	0.560*	0.703**	−0.143	0.077
S	Simpson	0.105	0.014	−0.336	−0.294	−0.203	0.133	0.133	0.231	−0.224	−0.392
	Chao1	−0.161	0.084	0.685*	0.671*	0.469	0.154	0.573*	0.420	0.217	0.517
	ACE	−0.161	0.084	0.685*	0.671*	0.469	0.154	0.573*	0.420	0.217	0.517
	Shannon	−0.168	0.098	0.531	0.601*	0.364	0.056	0.615*	0.371	0.140	0.559

^*a*^ Soil samples from 0 to 5 cm, 5 to 25 cm, and 25 to 50 cm depth were labeled as T, M, and S, respectively. *P < 0.05 and **P < 0.01.

Variations in the effects of soil characteristics on bacterial communities were observed across soil layers (Table 3). Specifically, SWC, pH, MBC, and NO_3_^−^ were identified as the most important factors contributing to variations in bacterial community in the T layer. In the M layer, the most important factors were SOC and ROC. In the S layer, the most important factor was SOC. The RDA results indicated that the soil factors could distinguish the soil bacterial communities across soil layers at our study sites (Fig. 6). Specifically, the largest influences on bacterial communities occurred with NO_3_^−^ and MBC, followed by pH, SWC, and ROC in the T layer. The first axis explained 39.61% of the variation in bacterial community, mainly driven by MBC, HEOC, DOC, and pH; the second axis accounted for 36.39%, mainly driven by SWC, NO_3_^−^, NH_4_^+^, and ROC. In the M layer, pH, SOC, and NH_4_^+^ had the greatest effect on the bacterial community. The first axis explained 74.04% of the variation, mainly related to SOC and DOC; the second axis explained 8.73%, mainly related to pH, CEOC, and MBC. In the S layer, the first axis explained 38.58% of the variation, primarily associated with pH, CEOC, and DOC; the second axis explained 28.99%, mainly related to NO_3_^−^, NH_4_^+^, and MBC.

**TABLE 3:** Results of partial Mantel test (*r* value) between soil bacterial community and soil characteristics across layers^*a*^

Soil characteristics	T (0–5 cm)		M (5–25 cm)		S (25–50 cm)	
	*r*	*P* value	*r*	*P* value	*r*	*P* value
pH	**0.319**	**0.025**	−0.027	0.459	0.011	0.439
SWC	**0.283**	**0.019**	0.188	0.085	−0.091	0.668
SOC	0.071	0.314	**0.413**	**0.026**	**0.480**	**0.012**
ROC	0.221	0.141	**0.609**	**0.003**	−0.021	0.485
MBC	**0.367**	**0.027**	−0.172	0.944	0.151	0.284
DOC	0.143	0.178	0.220	0.081	0.206	0.112
HEOC	0.104	0.263	−0.019	0.420	0.082	0.215
CEOC	−0.018	0.472	−0.005	0.482	0.210	0.162
NH_4_^+^	0.014	0.418	0.250	0.180	−0.039	0.585
NO_3_^−^	**0.390**	**0.011**	0.228	0.159	−0.069	0.580

^*a*^ The correlation and significance were determined between bacterial community (Bray–Curtis distance) and soil characteristics (Euclidean distance) based on 999 permutations. Significant correlations with *P* < 0.05 are shown in bold.

### Co-occurrence modeling of networks

**Fig 4:**
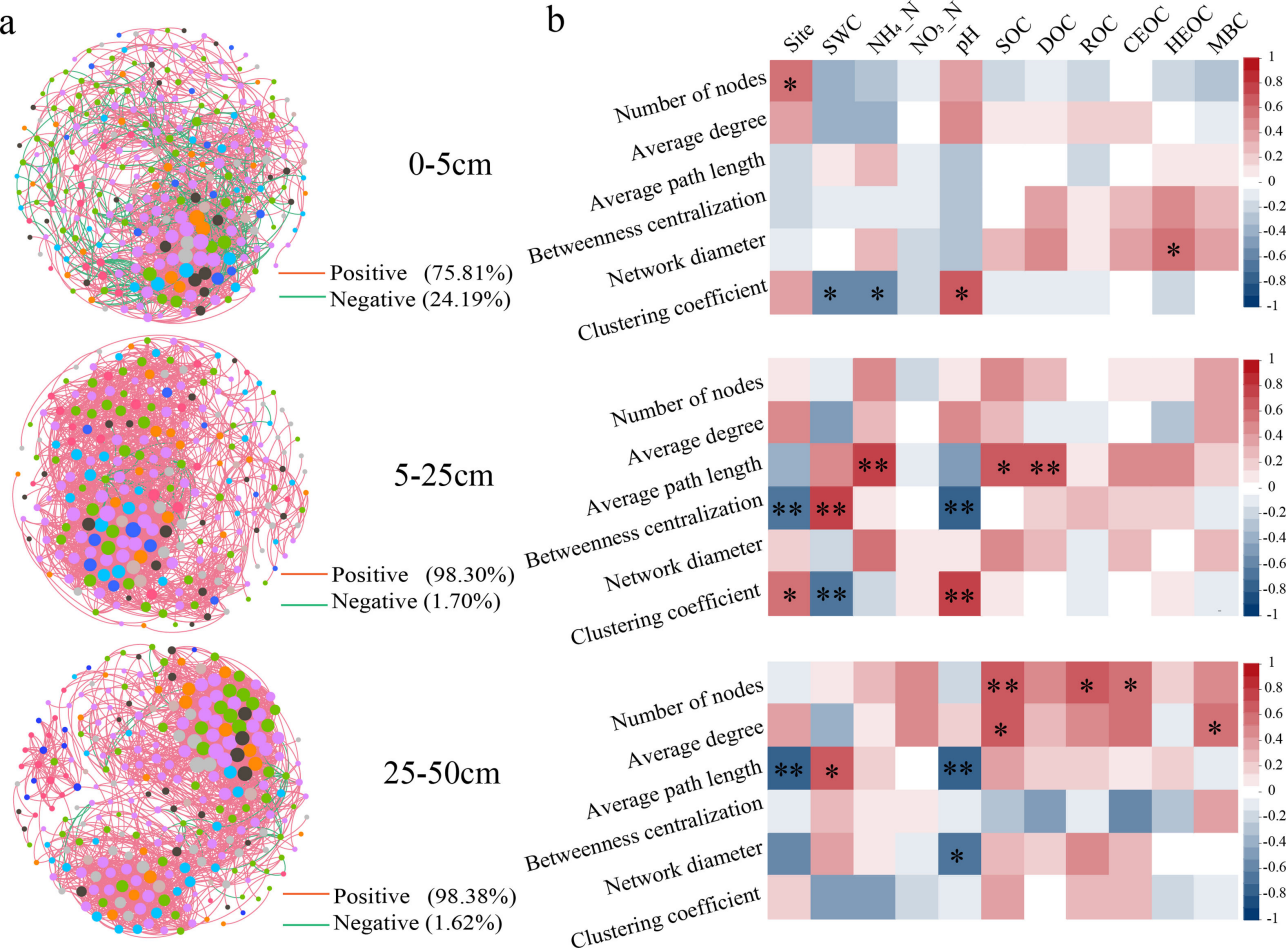
Co-occurrence network analyses of bacteria across soil layers. Correlation coefficients (*r*) with statistical significance (*P* < 0.05) and an absolute value over 0.7 were retained in the network analysis (a). Linkage between bacterial co-occurrence network topological parameter and soil properties (b). *P < 0.05, **P < 0.01.

Network analysis was used to analyze symbiosis patterns to explore the potential role of bacterial interactions across sites and soil layers. Symbiotic networks of soil bacterial communities varied distinctly across sites on topological properties (Fig. 4; also see Table S2 at https://doi.org/10.6084/m9.figshare.28255817.v1), as well as across soil layers on symbiotic networks and topological properties (see Table S3 at https://doi.org/10.6084/m9.figshare.28255817.v1). The 0–5 cm soil network had 1,769 edges with 1,341 (75.81%) positive and 428 (24.19%) negative links, whereas the 5–25 cm soil network included 2,371 (98.30%) positive and 41 (1.70%) negative links, and the 25–50 cm soil network consisted of 2,616 (98.38%) positive and 43 (1.62%) negative links (Fig. 4a). Also, number of nodes, average path length, network diameter, and clustering coefficient significantly varied across soil layers (see Table S4 at https://doi.org/10.6084/m9.figshare.28255817.v1). The results of Spearman’s correlation analysis revealed variation across the topological characteristics of the soil network with respect to the correlation of environmental variables (Fig. 4b). For instance, in the T-layer soil, the clustering coefficient was negatively correlated with soil SWC and NH_4_^+^, and positively correlated with soil pH. In the M-layer soil network, clustering coefficient was negatively correlated with NH_4_^+^, but the correlation was not significant (*P* > 0.05) (Fig. 4b).

**Fig 5:**
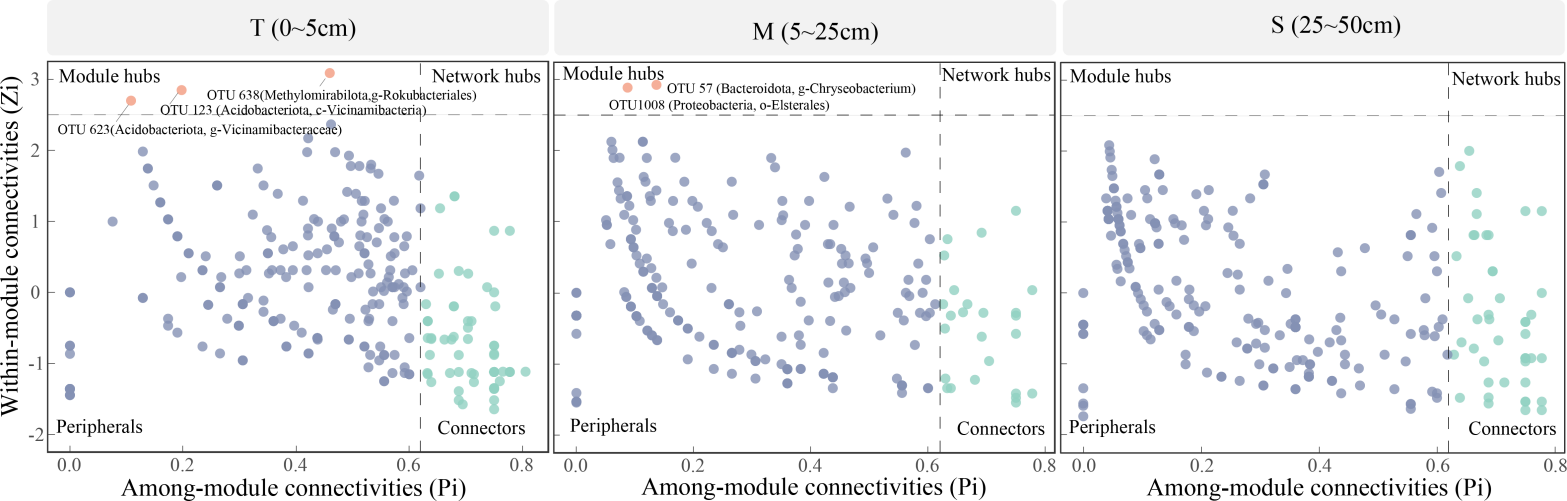
Zi-Pi plots showing the distributions of OTUs based on their topological roles in the 0–5 cm, 5–25 cm, and 25–50 cm soil layer networks. The threshold values of Zi and Pi used to categorize the OTUs were 2.5 and 0.62, respectively.

Using the Zi-Pi relationship analysis, we identified the key taxa of the ecological network by node classification (Fig. 5). Accordingly, all nodes were categorized into four parts: peripherals, module hubs, network hubs, and connectors, among which the majority were peripherals of the three soil layers. Generally, modular hubs and network hubs were recognized as keystone species. Notably, no network hubs were present in all soil layer networks in our study area. Instead, we identified multiple modular hubs (three nodes in the T layer network and two nodes in the M layer network), mainly belonging to the Phylum Acidobacteriota Class Vicinamibacteria (OTU123); Phylum Acidobacteriota Genus Vicinamibacteria (OTU623); Phylum Methylomirabilota Genus Rokubacteriales (OTU638); Phylum Proteobacteria Order Elsterales (OTU1008); Phylum Bacteroidota Genus Chryseobacterium (OTU57).

### Variation partitioning

The spatial factor explained the variation in the bacterial community across soil layers (see Fig. S6 at https://doi.org/10.6084/m9.figshare.28255817.v1). In the T layer, the results of variance distribution showed that the total explainable variance of the bacterial community was 38%, and the undetermined variance was 62%. The soil and spatial factors explained 23% and 7% of the variance, respectively. Soil and spatial factors collectively explained 8% of the variation in the soil bacterial community. In the M layer, the distribution of variance showed that the total explainable variance of the bacterial community was 41%, and the uncertain variance was 59%. Soil and spatial factors explained 34% and 4% of the bacterial community variation, respectively. In the S layer, the distribution of variance showed that the total explainable variance of the bacterial community was 21%, and the uncertain variance was 79%. Soil and spatial factors explained 13% and 6% of the bacterial community variation, respectively. Notably, spatial factors explaining variation in bacterial communities decreased with soil depth.

## DISCUSSION

### Bacterial diversity and community composition across soil layers

Our results revealed that soil bacterial α-diversity and community structure varied considerably in the same soil layer within the northern mountainous area of Longmenshan, which did not support our first hypothesis. Soil properties and space attributes significantly affected the composition of the belowground communities in different sites at a small scale. Bacterial diversity generally decreased with soil depth (4, 10), probably due to the fact that soil nutrients (e.g., C, N, and P) decrease with soil depth, affecting soil microbial diversity (34–37). Notably, soil bacterial richness varied significantly across soil layers and decreased obviously from surface to subsurface soils in our study, which is consistent with the results of Song et al. (4). Whereas the diversity index varied nonsignificantly with soil depth, which could be attributed to consistent vegetation types and diversity at our sampling sites. Previous studies have confirmed that bacterial diversity mainly correlates with the plant Shannon index, and bacterial variability can reflect changes in plant communities and soil properties (4, 36, 38).

Bacterial diversity and composition varied considerably across soil layers. For example, we found that the dominant bacterial group in the surface layer was Proteobacteria, which accounted for 35.28%; the middle layer accounted for 32.62%, and the bottom layer accounted for 29.47%. Significant correlation between Proteobacteria and SOC has been confirmed (12, 15, 16, 39), indicating a strong impact of plant litter decomposition products and root secretions on their growth. Despite the small scale in our study area, bacterial community varied considerably across sites and soil layers. Nutrients were negatively or positively correlated with the relative abundance of bacteria, which to some extent supports our conclusion (see Fig. S2 and S3 and Table S1 at https://doi.org/10.6084/m9.figshare.28255817.v1). Dominant taxa of bacteria at the phylum level across soil layers were Proteobacteria and Acidobacteria in our study. This is possibly due to the fact that their adaptability enables them to survive in various environments (37, 40). The bacterial indices decreased with soil depth, possibly due to a reduction in the nutrient substrate (34–37). Differences in microbial diversity were not significant at the regional scale, whereas variations in richness were greater, and those variations may be related to nutrient variability.

### Bacterial community drivers in different soil layers

**Fig 6:**
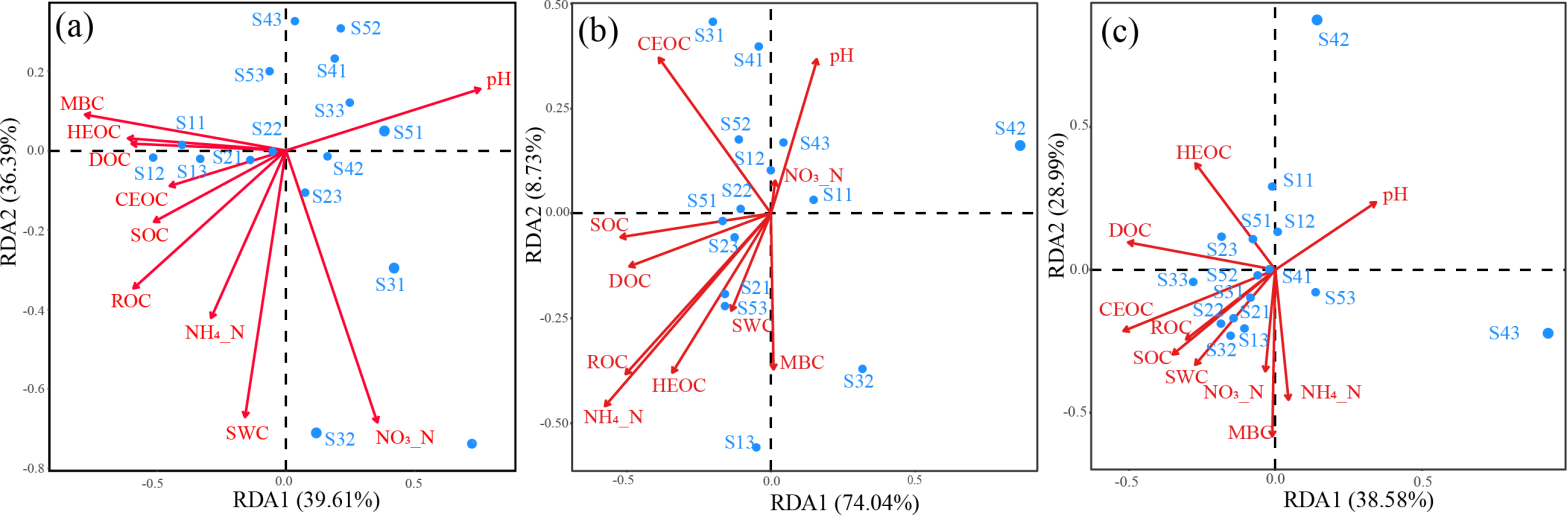
Redundancy analysis (RDA) between soil chemical properties and soil bacterial communities at OTU level in T (a), M (b), and S (c) layers.

Soil properties, as a critical determinant of soil microbial communities, have been recognized for a long time (4, 39). Bacterial community composition was found to be mainly correlated with SWC and pH in surface soils, whereas correlated with SOC and its fractions in subsurface soils. Previous studies and meta-analyses also assessed the effects of pH and SWC on microbial diversity and community structure (26, 37, 41–43), and the results of these studies suggest that soil pH and SWC represent important predictors of microbial α-diversity to global change factors (36, 39, 43–47). Our study identified that bacterial diversity correlated positively with pH and negatively with SWC in topsoil (Table 2). Based on partial Mantel test and RDA analysis, pH and SWC dominated the variation in bacterial community structure of topsoil (Fig. 6a and c; Table 3). Probably, soil pH and SWC influence changes in community structure through environmental factors (e.g., nutrient effectiveness and organic carbon content), which tended to change simultaneously with variations in soil pH and SWC (5, 39, 48). Also, pH and SWC can impact vegetation populations and soil animals (5, 39), which lead to altered soil properties and thus affect the soil microbial community. The absence of acidic sites in our study region may partially reduce the effect of soil pH on microbial community.

Spearman’s rank correlation analyses and partial Mantel tests showed that SOC and its components (such as ROC, HEOC, CEOC, and DOC) represented the main factors driving bacterial diversity and community structure in deeper soils (Tables 2 and 3). Compared to topsoil, the deeper soils may more likely be anoxic, where microbes tend to grow anaerobically, and thus affect the microbial community composition. Bacterial richness and diversity of deep soil were correlated positively with SOC and its fractions (Table 2; also see Table S1 at https://doi.org/10.6084/m9.figshare.28255817.v1). Traditional ecological niche theory holds that different species coexist in ecosystems by occupying different ecological niches; carbon (C) substrate effectiveness may be restricted in soils where SOM content was very low (21, 49), and the ecological niches of many species may be related to C effectiveness (35, 50, 51). Besides, Bacteroidia was positively correlated with SWC, whereas Gemmatimonadetes and Blastocatellia were negatively correlated with SWC in the M layer; α-Proteobacteria was positively correlated with SWC, whereas Methylomirabilia was positively correlated with SWC in the S layer (see Fig. S4 at https://doi.org/10.6084/m9.figshare.28255817.v1). Overall, soil microbial diversity and community structure variations across soil layers were driven by different soil properties, which confirmed our hypothesis.

### Bacterial network properties and keystone species varied across soil layers

Network analysis enabled us to identify previously unnoticed patterns of microbial symbiosis so as to decipher the complex relationships between microbial taxa (4). Results revealed that number of nodes, average path length, and network diameter of the surface soil network were larger than those of the subsurface soil, and this was consistent with our third hypothesis. The number of positively correlated edges was considerably larger than the number of negatively correlated edges with soil depth, and we inferred that the symbiotic relationship of the bacterial community was more obvious (52). It also indicated that the bacterial community in Longmenshan showed a larger effect on the variation of environmental factors with soil depth. Network topological parameters can characterize the complexity of community network relationships. Typically, network complexity correlated positively with microbial community stability. Small path-length networks are recognized as small-world networks, which are associated with rapid ecosystem responses to disturbances (4, 53). The subsurface network was more complex than the surface network but was less correlated with soil variables (Fig. 4; also see Table S4 at https://doi.org/10.6084/m9.figshare.28255817.v1). It also indicated that the bacterial community in Longmenshan had more influence on the variation of environmental factors with soil depth (31). Soil layers had a larger effect on the bacterial symbiotic network and topology than the site in this region (see Table S5 at https://doi.org/10.6084/m9.figshare.28255817.v1). This is probably due to the small scale in our study area and nutrient content variation across sites less than that in different soil depths.

Keystone species perform a crucial role in maintaining ecological community structure and affect the community more than expected based on either their relative abundance or gross biomass (4, 54). Our study also revealed that the critical nodes of the co-occurrence network varied across soil layers. The most dominant key species taxa include Phylum Methylomirabilota Genus Rokubacteriales, Acidobacteriota Class Vicinamibacteria, and Phylum Acidobacteriota Genus Vicinamibacteria in surface soils. In M soils, the dominant OTUs include Phylum Proteobacteria Order Elsterales and Phylum Bacteroidota Genus Chryseobacterium, which perform important soil functions in the Longmenshan. Notably, there is an absence of key species taxa in subsurface soil. Acidobacteria are commonly regarded as oligotrophic microorganisms, preferring more nutrient-poor habitats and specializing in the decomposition of difficult-to-degrade soil carbon (4, 55). Also, the bacterial networks had different key taxa across soil layers, which further evidenced ecological niche differentiation among bacterial taxa along the soil depth. Although we have found that keystone species varied across soil layers, the specific ecological roles of these keystone species are still unknown. Hence, further investigation of the roles and functions of soil keystone species in ecosystems would be critical for research on soil health, such as soil restoration and fertility maintenance.

### Relative contributions of soil and spatial factors to bacterial community across soil layers

Soil properties and spatial factors were important factors resulting in variations in bacterial communities across soil layers in our study (see Fig. S5 at https://doi.org/10.6084/m9.figshare.28255817.v1). Previous studies have shown that soil properties and spatial structure also provide a major influence on microbial communities (37, 56–58). Compared to spatial structure, soil properties contributed most to bacterial communities across soil layers (5, 59), thus confirming our hypothesis. Also, some studies have shown that geographic distance explains microbial community shifts at the regional scale (5, 33). ADONIS and ANOSIM analyses showed obvious differences in the bacterial community across sites within the same soil layer, as well as significant variations across soil layers (see Table S2 at https://doi.org/10.6084/m9.figshare.28255817.v1), which showed discrepancies with Kang et al. (5). Probably due to the fact that we selected mountain soils for our study, whereas they selected plateau wetlands for their study. Distinct role of environmental factors in the microbial community can further explain obvious bacterial shifts across spatial distances and soil depths. Our study also indicated that due to diverse responses of bacteria to environmental factors, bacteria exhibited varied metabolic separation and resource preferences in the soil within even short distances.

Notably, we chose only a relatively small study area in the northern mountainous region of Longmenshan, within a total of 5 sites and 15 soil profiles. The objective of this approach was to minimize the influence of climatic factors on the study as much as possible, with a view to obtaining more reasonable results. Besides, the comparatively small sample size probably affected the reliability of our results. Instead, more samples could provide more details, which could lead to a better insight into the spatial distribution characteristics of soil microbial communities and their drivers at small regional scales. Although our results are not universally generalizable, it can be confirmed that our results could serve as a complement to the global pattern of vertical distribution of soil microorganisms in mountainous areas.

### Conclusion

The results indicated that pH and SWC were the key factors affecting bacterial diversity and community structure in the surface soil layer. Soil surface bacterial diversity showed a significant positive correlation with pH and a negative correlation with SWC. Bacterial networks varied significantly across soil layers. Bacterial networks differed significantly across soil layers and varied differently with changes in soil environmental variability. Soil depth had a clearly greater effect than spatial effect on soil bacterial community. Environmental factors (explaining 13%–34% of the variance) and spatial factors (explaining 4%–7% of the variance) accounted together for most of the bacterial community variation. These results indicated that soil factors represent the main drivers of microbial community shifts, but spatial factors also played a role in microbial community composition that cannot be ignored, even in the small-scale region. We also revealed specific key species of bacterial communities across different soil layers, demonstrating the differential and complex response of microorganisms to environmental changes in mountain ecosystems. Future studies should focus on priority taxa and their functions in the ecosystem. The knowledge of the ecological functions of the soil is important for maintaining the quality of soil and agricultural products.

## Data Availability

Relevant data supporting the critical findings of this study are available within the article. 16S amplicon sequences were deposited in NCBI under BioProject accession number PRJNA1218530.
